# Bright’s Disease

**Published:** 1882-10

**Authors:** Alonzo Clark

**Affiliations:** Professor of Pathology and Practice of Medicine, College of Physicians and Surgeons, New York


### Selections

### Bright's Disease.

By Alonzo Clark, M. D:,

Professor of Pathology and Practice of Medicine, College of Physicians and Surgeons, New York.

First patient, a male, about fifty years of age. He states that he has been confined to the house about four months; that he has to stagger from one place to another across the floor, but that he is better now. The feet are swollen; much less, however, than they were previously. He prefers to lie on the left side at night, which may account for the fact that the left leg is swollen more than the right. The leg pits deeply. He also complains of having pain in the back. This symptom, with the weakness in the limbs which he complains of on walking, would suggest spinal disease I have known persons with the legs considerably swollen. They could walk well enough, but when they got on the bed they could not lift their legs up. This patient says such has been his case also. That, probably, is the explanation of his awkward movement, especially as he has had no paralysis anywhere else. It is true he thinks the sensation in his legs is considerably diminished, but when I pinch him he grumbles.

Then the question is, what is the cause of the swelling of his legs? That is the most marked feature of his case. The answer naturally is, kidney disease; but Dr. Wheelock informed me that he examined the urine and did not discover albumen. That may be; for persons sometimes come to me saying they have Bright's disease, been so called by their family physician, and I examine the water and find no albumen. I do not conclude that they have not Bright's disease, but continue to examine it every second or third day for three or four weeks, because I know the microscopic, the chemical and the clinical symptoms are often absent for two or three weeks in a case that is really confirmed, and then will come again. The fact, therefore, that no albumen has been found in the water is by no means conclusive, especially when we have the evidence of the œdema. The œdema has been confined mostly to the legs and feet; he says he has not had any swelling of the hands.

There is an early sign of coming œdema that is, however, fallacious as a distinctive sign. I refer to the tear line. A little œdema will come on the conjunctival membrane of the lower lid, and pressing the lid against the eye, will force it up on to the top of the lid, and it will then have a little wire appearance, or the appearance of a small thread running along the lower eyelid. This patient has that. There is a little œdema of the conjunctiva elsewhere also. It goes away when you lift the lid, but comes again immediately after. He says there has been no enlargement of the abdomen.

He has had trouble in going up stairs during the last week or two, but it seems to have been due principally to the condition of his legs. He says he does not get out of breath except on some exertion.

We will examine his chest and abdomen. The fact that he prefers to lie on the left side is rather evidence against cardiac disease. Persons who have much of that trouble are very apt to lie upon the right side or back. I do not get any signs of valvular lesion about the heart, although there is a little hypertrophy of that organ. There is no pain in the chest. There is dullness behind, over the lower portion of the thoracic cavity, indicating a little fluid in either pleural cavity. There is, you will observe, some œdema of the face and swelling under the eyes.

I think this is an instance in which it is safe to infer that there is some trouble with the kidneys, and that, at the present time, conclusive evidences are not apparent, but perhaps a week hence it will be different.

With regard to the treatment in such a case, the great point is to keep himself warm. If he is obliged to go out in cold weather, he should protect himself with an abundance of clothing. If he is not obliged to go out, it is better, in cold weather, to keep in the house. I have kept such patients in the house all winter, and directed them to keep the temperature of their part of the house at 72° F., night and day. Not so particular at night, because the bed-clothes will protect them, but it is better to have it uniform. This man cannot do that; he will have to protect himself as much as he can in cold weather by clothing. A steam bath is a very excellent thing, to be taken twice a day, and anybody can arrange a home apparatus for that purpose. It may be prepared by taking a tin can that will hold a couple of gallons of water, covered with some utensil; put a tube in the dome of it; connect with this tube another one, six feet long, having an angle, which will convey the steam to the wooden chair on which the patient sits while covered with blankets, fastened about the neck. In a few minutes the patient will begin to perspire, and this may be kept up say twenty minutes or half an hour, when he should be well rubbed off, and if it be in the evening, as I generally direct, he should put on his night clothes and go to bed. If he sweats a little after that, all the better. Then in the morning before he gets out of bed, he should be rubbed thoroughly with a dry flannel so as to stimulate the circulation. That stimulation of the circulation of the skin with dry flannel or some agent that is equal to it should be practiced every day, whether he takes the bath or not the night before. I was in consultation near here some years ago, and it was agreed that to sweat the patient was desirable, and we were devising a plan by which it could be carried out when the nurse asked what was wanted. It being explained to her, she said she could do that. Boil a peck of potatoes; wrap them in a cloth and lay them close to the body. She did so, and it worked very well. It is often the case that bottles of water, not exactly hot but pretty nearly, are enveloped in damp cloths and laid alongside the body. The œdema will sometimes disappear on the use of these steam baths, but commonly it is necessary that some diuretic be used to accomplish that. All effusions that are observable in the body are easiest carried off by the kidneys, disturbing the system less than if carried off by any other emunctories that are capable of doing it.

For him, probably, as good a diuretic as any that can be prescribed is the sal diureticus, the acetate of potash, twenty grains every two hours, in water; and the infusion of digitalis, commonly a dessert spoonful, or two drachms, will be enough, taken three times a day. He cannot conveniently take it with the salt, because he takes that every two hours.

### FACIAL PARALYSIS.

*Case 2.*—This man complains of a disfigurement of the face. He works by night and sleeps by day, and about two weeks ago, having gone to bed perfectly well, he awoke, and observed that the right side of the face was paralyzed. The right eye watered. No pain was felt, but the face appeared disfigured.

This kind of deformity, paralysis, often comes of inflammation of the theca of the seventh nerve, the portio dura, and in that case leeches are the quickest remedy that we can use, applied as near to the source of the nerve as can be done; but in this case there is no tenderness at all. I press pretty hard, to the extent of a couple of pounds weight, and he says it does not hurt him any. This is not a very uncommon affection, and it generally comes just as in this man's case; it comes in the night, and I think it is more likely to occur if there is a draft of air upon the side of the face corresponding with the paralysis. Many of these cases will be relieved without any remedy in the course of four, five or six weeks. In others it is of very considerable duration; indeed, in some it is not relieved at all, being carried for years. Relief, when it is effected, is obtained from various sources; sometimes from electricity. Medicine, I think, has no power over it at all; at least I know of none As just said, electricity seems to be remedial in certain instances. Dr. Detmold, some years ago, invented a little electrical apparatus which he thought would act well in these cases, and I think it does. It is a compound metal wire, containing two metals that act upon each other in a way to produce electricity. At one end is a hook which goes in the mouth, lifting the angle of the mouth up on the affected side, and at the other end is a hook that goes over the ear. He has the patient wear that continually, except while eating. It is a simple apparatus, and the saliva of the mouth probably aids in making it electrical.

You observe, as the patient smiles, how the face is drawn to the left side. There is no affection of the tongue that I can see. He can chew his food all right on either side. Sometimes the paralysis is so positive that the food will come out of the mouth when he chews with the teeth of the affected side, but it does not seem to do so in this man's case. I see some movement of the muscles of the paralyzed side, as he talks, which seems not to be entirely passive.

### SPINAL SCLEROSIS.

*Case 3.*—Our next patient is a man about thirty-three years of age, who, as you see, has been led into the room by his wife. She says he has been sick about two years; has not been able to walk for about a year and a half. He has cramps; sometimes unconscious four or five hours after the attack. In the attacks the arms and legs are straightened and shake. The hands are clinched, and he attempts to get out of bed; talks, but appears to be unconscious. After the attack he asks what has been the matter, remembering nothing that had occurred.

Some of these symptoms point to epilepsy, while others do not. It is not uncommon that persons after an epileptic attack are really delirious, sometimes for half an hour, sometimes for four, five, or seven hours. She says he is white during the attack, which would not point to epilepsy.

Some days he cannot walk at all, and you will observe that muscular paralysis is plain enough. The next point arises, is there paralysis of sensation. Not being provided with a special instrument to test the sensation, we will take a card that will hold firmly pins stuck through it, putting the pins two or three inches apart, to begin with, and reducing the distance gradually as we test his sensation.

You will observe that there is no defect in the sensation in his case. We would infer that there is here thickening of the posterior column of the spinal cord and not of the anterior. This is not a very uncommon form of sclerosis. He has not the walk of locomotor ataxia, but his wife's description of his walk formerly corresponds to the walk of that affection. We may infer, then, that he really had locomotor ataxia, and that it is now being converted into paralysis. We have such a patient in the hospital at present, who was treated in a variety of ways, by medicines given internally, until we finally settled down upon the fluid extract of ergot, increasing the dose until he got to taking a drachm three times a day. He continued that an entire year, at the end of which time he was so far restored that he could take the place of orderly about the wards. He has now been in the hospital five years, and some of the professors show him to the students every year.

Now, as an encouragement in this case, I should think the same medicine would give some relief to this man. Dry cups might also be applied over the spine, as it can be done by some one of the family, by burning a piece of paper in a small-sized tumbler, placing four on the back, two on either side of the spine. This, with the fluid extract of ergot three times a day, would be the treatment. I would not start out with a drachm of the medicine, but half a drachm, and see if he bears it fairly. This reminds me that we now have in the hospital a man suffering from dry gangrene of the toe. The pulse is good, and there is no obstruction in the arteries. We learned, after he had been in the hospital a day or two, that he had been eating rye bread; that for years he had eaten it whenever he could get it, and living in the West he could generally get it easily enough. The question presented itself before us whether this dry gangrene was the result of ergot which he got in the rye. In times past, when rye was used a great deal, dry gangrene occurred, which was attributed to the rye, not, perhaps, to ergot which it contained, for that was little known until about the time of Dr. Stearns. But I am not sure about the agency of rye bread in this case of dry gangrene of the toe.

### GRAVES' DISEASE.

*Case* 4.—Here is a case that you will be interested in. You will observe, first, that there is swelling in the neck. I have not seen her before, but the case struck me in an instant as she entered. Second, there is some degree of prominence, not remarkable, of the eyes. And third, I suppose we shall find the pulse beat at the rate of 120, perhaps beating more rapidly than under ordinary circumstances, coming into a room where there are so many strange faces. But I do not find any pulse beat at all, either at the wrist or elbow. The heart beat, however, is strong enough, and exactly at the rate of 120. The hands are warm enough; I do not know why the pulse cannot be felt.

She has, then, three elements of what is sometimes called Graves' disease, because he described it before anybody else, in the English language. It is claimed for Basedow in Germany, and for Trousseau in France, but Trousseau acknowledges that Graves described it before, so that it is not very wrong to call it Graves' disease. The thyroid gland is swollen, the right side

9

more than the left, in this case. It is a mild case, showing the three characteristic features of it in a limited degree only. Congestion produces these swellings in the thyroid gland and behind the eye. How it should fall out that three parts of the body that are at a considerable distance from each other should undergo the same change, and the intermediate parts not be affected, is a matter difficult to explain. Still, case after case presents itself, though it is a rare affection, and we are obliged to assume that Graves was right in associating the three congestions (he did not explain it, however), and that it makes really one disease.

The patient says her general health is good; she eats well, digests well, has no pain. She does her housework and thinks she is as well as usual.

You ask what I would prescribe? That same fluid extract of ergot, because of its effect upon the capillaries. I would have her begin with twenty drops, increase it to thirty, forty, and perhaps to fifty, in a week or ten days, and if it works as well as it has done in some of the cases I have seen, she will be improved in a moderate length of time. Speaking from my own experience, it always gets well; but I notice in the records of other persons it does not always. At any rate, there is hope for improvement in this case, as it is not a very well developed one. It has these three features, but none of them marked in a high degree.—*Medical and Surgical Reporter.*

